# Serological and molecular investigation into the role of wild birds in the epidemiology of West Nile virus in Greece

**DOI:** 10.1186/1743-422X-9-266

**Published:** 2012-11-12

**Authors:** George Valiakos, Antonia Touloudi, Labrini V Athanasiou, Alexios Giannakopoulos, Christos Iacovakis, Periklis Birtsas, Vassiliki Spyrou, Zisis Dalabiras, Liljana Petrovska, Charalambos Billinis

**Affiliations:** 1 Laboratory of Microbiology and Parasitology, Faculty of Veterinary Medicine, University of Thessaly, 224 str. Trikalon, Karditsa, 43100, Greece; 2 Laboratory of Zoonoses Research, Institute of Biomedical Research and Technology (BIOMED/CERETETH), Larissa, Greece; 3 Department of Medicine, Faculty of Veterinary Medicine, University of Thessaly, Karditsa, Greece; 4 Department of Forestry and Natural Environment Administration, Technological Education Institute of Larissa, Karditsa, Greece; 5 Hunting Federation of Macedonia and Thrace, Thessaloniki, Greece; 6 Department of Animal Production, Technological Education Institute of Larissa, Larissa, Greece; 7 Department of Bacteriology, Animal Health and Veterinary Laboratories Agency, Weybridge, UK

**Keywords:** Greece, Molecular epidemiology, Outbreak, Serological surveillance, West nile virus, Wild birds

## Abstract

**Background:**

A West Nile virus (WNV) disease outbreak occurred in 2010 in northern Greece with a total of 262 laboratory-confirmed human cases and 35 deaths. A serological and molecular surveillance was conducted on samples of hunter-harvested wild birds prior to and during the outbreak.

**Findings:**

Serum and tissue samples from 295 resident and migratory wild birds, hunter-harvested during the 2009–2010 and 2010–2011 hunting seasons at the epicenter of the outbreak in northern Greece, were tested for the presence of WNV-specific antibodies by immunofluorescence assay and virus neutralization test. WNV neutralizing antibodies were detected in 53 avian samples. Fourteen positive sera were obtained from birds hunter-harvested up to 8 months prior to the human outbreak. Specific genetic determinants of virulence (His249Pro NS3 mutation, E-glycosylation motif) were recognized in a WNV lineage 2 strain isolated from a hunter-harvested Eurasian magpie and a nucleotide mismatch was revealed between this strain and a mosquito WNV strain isolated one month earlier in the same area.

**Conclusions:**

This is the first report regarding exposure of wild birds to WNV prior to the 2010 outbreak, in Greece. Results provide evidence of the implication of wild birds in a local enzootic cycle that could allow maintenance and amplification of the virus before and during the outbreak. Findings of past exposure of migratory birds to WNV upon their arrival in Greece during autumn migration, suggest avian species with similar migration traits as candidates for the introduction of WNV into Greece. The possibility that an endemic circulation of WNV could have caused the outbreak, after an amplification cycle due to favorable conditions cannot be excluded.

## Background

West Nile virus (WNV) is a flavivirus of major public health concern for the last 2 decades, as associated disease outbreaks are increasing worldwide. The main route of infection is through the bite of infected mosquitoes; humans and horses develop viremia levels of low magnitude and short duration, insufficient to re-infect mosquitoes, and thus do not serve as amplifying hosts for WNV in nature [1].

On the contrary, various avian species develop viremia levels sufficient to infect mosquitoes and even bird to bird transmission of the virus by direct contact has been reported [2]. Hence, WNV is maintained in an enzootic cycle with birds being the amplifying hosts and ornithophilic mosquitoes, especially of the *Culex* species, the main vectors. Moreover, local movements of resident birds and long-range travel of migratory birds may both contribute to the spread of WNV [3,4].

In 2010, a major outbreak of WNV human infections occurred in northern Greece, with 262 laboratory-confirmed cases and 35 deaths [5]. Although WNV neutralizing antibodies had been detected in northern Greece since 2007, the first WNV lineage 2 (L2) strain was obtained from pools of Culex mosquitoes (strain Nea Santa-Greece-2010) in 2010 [6]. At the same time our team detected a similar L2 strain in a Eurasian magpie (strain magpie-Greece/10), as has been reported [7].

For the purposes of our participation in an FP7 EU research project (“WildTech”), wild birds samples that have been collected by the Hunting Federation of Macedonia and Thrace since 2009 were used for serological and molecular surveillance regarding exposure to various pathogens. The objective of this study was to detect possible exposure of wild birds to WNV prior to and during the outbreak. In addition we further investigated the detected WNV magpie strain for important virulence markers. These markers have been recognized to be a prerequisite for the development of viremia levels in wild birds necessary for them to be considered amplifying hosts.

## Findings

**Figure 1:**
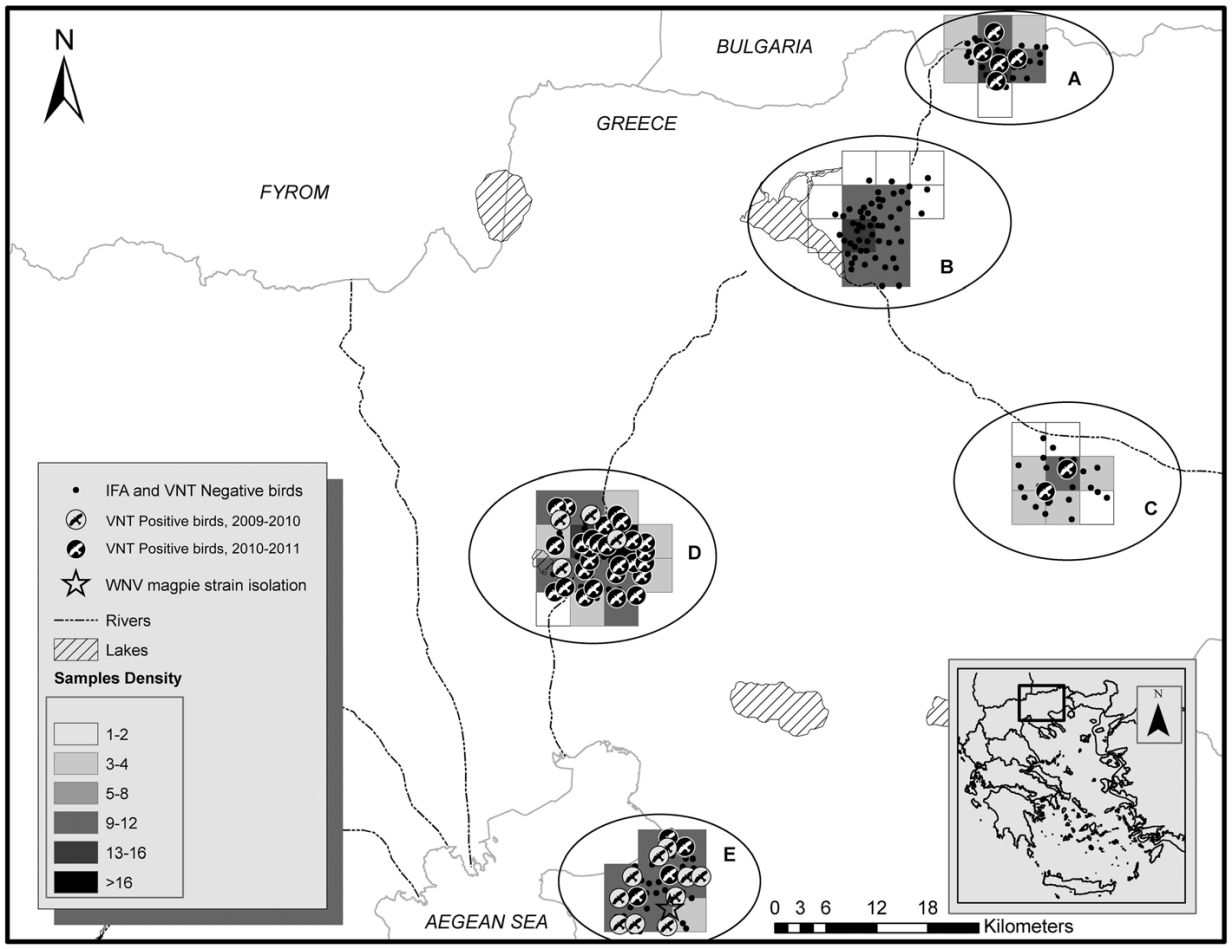
**Map of the epicenter of the WNV outbreak, Greece 2010.** Map of the epicenter of the outbreak where most of the human West Nile Virus cases occurred during 2010 (Central Macedonia, Northern Greece). The area is characterized by wetlands and is considered a well-known resting and breeding area for migratory birds. Sites A-E refer to bird sampling sites.

Our team conducted a serological and molecular surveillance in serum and tissue samples of wild birds hunter-harvested in 2009–2010 and 2010–2011 official hunting seasons (from 20 August until 28 February of the following year). Samples were collected at the epicenter of the 2010 outbreak (Figure 1) in central Macedonia by members of the Hunting Federation of Macedonia and Thrace from species considered quarry according to Greek legislation. Samples from all different species were not available for both periods or from every sampling site. All sampling sites are in flying distance of avian species and no safe conclusions can be drawn regarding viral dispersion in the area between the hunting seasons. This area is characterized by mosquito-abundant waterlands and four major rivers which converge into a common delta, a well-known resting and breeding ground for migratory birds. The study was focused on hunter-harvested resident and migratory avian species suspected to play a role in WNV local circulation, maintenance and dispersion. Members of the *Corvidae* family, like Eurasian magpies (*Pica pica*) and hooded crows (*Corvus cornix*) were prioritized; these corvid species are resident, with a wide daily dispersal range of up to 20 km, social, roosting in large colonies and abundant in both wetlands and urban areas [8]. Turtle doves (*Streptopelia turtur*) were also targeted; they are suspected to be a principal introductory host of WNV via their migration routes, as the virus has been isolated from actively migrating turtle doves [9]. Migratory waterfowl like the mallard ducks (*Anas platyrhynchos*) have been found to carry WNV antibodies and recent experimental studies have also proven that *Anseriformes* may be able to function as carriers of WNV [10].

Serum and tissue samples from 295 hunter-harvested birds belonging to the above 4 avian species were collected. Serological screening was performed with an indirect immunofluorescence assay (IFA) test kit (EUROIMMUN®) with slight modifications of manufacturers’ instructions, to detect avian WNV antibodies, as previously described; the determined cut-off value of 1:30 was used and a (FITC)-labeled goat anti-bird antibody was applied (Bethyl Laboratories Inc.) [11]. Positive results were verified by a micro-virus neutralization test (VNT), as previously described [12].

Serological results are summarized in Table 1 and VNT titers are presented in Table 2. Seventy samples (23.7%) were IFA-positive, 53 of the IFA-positive samples were confirmed by micro-VNT test. WNV-neutralizing antibodies were detected in 14 resident corvid samples hunter-harvested in the 2009/2010 hunting season; six seropositive corvids were collected in October 2009, indicating an avian exposure to WNV at least 8 months prior to the human outbreak. Presence of WNV-neutralizing antibodies in corvids sera collected in both 2009/2010 and 2010/2011 hunting seasons indicates a continuous maintained circulation and presence of WNV in the area for the referred period, as hunter-harvested corvids are mainly < 1 year old, born in February-March of the same year.

**Table 1:** Summary of West Nile virus serological results among hunter-harvested wild birds, Greece 2009-2011

**Species**	Sampling site*	**Total no. of bird samples**	**Hunting season 2009-2010**		**Hunting season 2010-2011**		**Total**	
			**IFA pos./ neg.**	**VNT pos. (n)**^†^	**IFA pos./ neg.**	**VNT pos. (n)**	**IFA pos./ neg.**	**VNT pos. (n)**
*Pica pica*	D	90	5/5	3 (5)	34/46	26 (34)	39/51	29 (39)
	E	58	10/24	8(10)	4/20	3 (4)	14/44	11 (14)
*Corvus cornix*	D	11	1/3	1 (1)	3/4	2 (3)	4/7	3 (4)
	E	12	3/5	2(3)	1/3	1 (1)	4/8	3 (4)
*Anas platyrhynchos*	B	55	0/25	-	0/30	-	0/55	-
*Streptopelia turtur*	A	45	-	-	6/39	5 (6)	6/39	5 (6)
	C	24	-	-	3/21	2 (3)	3/21	2 (3)
Total		295	19/62	14 (19)	51/163	39 (51)	70/225	53 (70)

Prevalence of West Nile virus antibodies among wild birds hunter-harvested at the epicenter of a major human outbreak of the disease (central Macedonia, northern Greece). All IFA positive samples were further verified by VNT.

*Sampling Sites A-E correspond to the indicated areas of Figure 1.

^†^n number of samples tested positive by IFA.

**Table 2:** Virus neutralization titers of avian sera samples considered positive

**Sampling site**	**Species**	**Number tested**	**1/10**	**1/20**	**1/40**	**1/80**	**1/160**
A	*Streptopelia turtur*	5	1	1	1	2	-
C	*Streptopelia turtur*	2	-	1	-	-	1
D	*Pica pica*	29	4	14	5	3	3
	*Corvus cornix*	3	-	-	2	1	-
E	*Pica pica*	11	3	6	-	1	1
	*Corvus cornix*	3	-	1	1	-	1

West Nile virus neutralizing antibody titers measured in VNT positive samples (threshold 1/10 for a sample to be considered positive) of each sampling site as indicated in Figure 1.

Seven sera of turtle doves hunter-harvested upon the days of their arrival in resting areas (sites A and C, Figure 1) during autumn migration were found positive for WNV-neutralizing antibodies. Three of these 3 were juvenile, born probably in breeding areas of origin in the same year (central Europe). None of the 55 sera of mallard ducks hunter harvested near the artificial Lake Kerkini, a premier birding site (site B, Figure 1), were found to contain WNV-specific antibodies.

Molecular screening from pools of selected tissue samples (liver, heart and kidney, known to be suitable samples for WNV detection especially in asymptomatic birds) was performed as described previously [7]. A positive WNV L2 PCR product was obtained from a magpie hunted in the area of the human outbreak in September 2010 that was, as reported, similar to the one derived from pools of mosquitoes in the same area [6] and showed the highest sequence similarity to strains derived from birds of prey in Austria in 2008–09 as well as in Hungary in 2004 [13,14]. In this study, further molecular investigation of the magpie WNV strain was performed, regarding important virulence markers. RT-PCR and a subsequent sequencing analysis was employed for the amplification of a 270-nt Envelope (E) protein genomic region, using previously described primers [15]. Molecular investigation of a 401-nt NS3 genomic region of the magpie strain using a previously established PCR protocol, was also performed [16].

**Figure 2:**
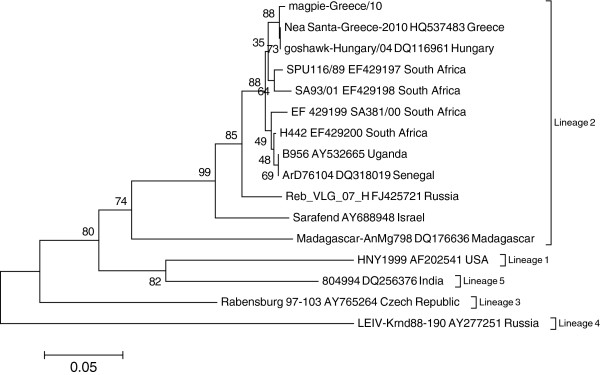
**Phylogenetic tree of the 270-nt E genomic region.** Phylogenetic analysis based on a 270-nt E genomic region of 16 West Nile virus strains (including strain of the present study, shown on top) was performed by using MEGA version 5. GenBank accession numbers and geographic origins of strains are shown. Neighbor-joining tree was constructed from a difference matrix employing the Kimura 2-parameter correction. One thousand bootstrap pseudo-replicates were used to test the branching (shown as percentages).

The N-linked glycosylation motif (N-Y-T/S) at residues 154–156 of the E protein is present in the magpie strain; E protein glycosylation is considered a prerequisite for the development of the necessary viremic levels in avian blood (> 10^5^ PFU/ml) that allow efficient transmission of WNV Lineage 1 from avian hosts to mosquitoes [17]. A nucleotide mismatch was revealed at nt position 624 of E gene (G-C transversion, synonymoys SNP) by pairwise alignment between the present study magpie WNV strain and the mosquitoes WNV strain isolated one month earlier [18]. The E genomic region sequence from the Greek magpie isolate was deposited in GenBank under accession no JN809470. A phylogenetic tree was constructed using MEGA 5.0 [19]. Neighbour-joining analysis of genetic distances in the 270-nt E genomic region of WNV strains (Figure 2) displayed close phylogenetic relationship between the Greek WNV strains, a Hungarian WNV strain isolated from a goshawk in 2004 and South African WNV lineage 2 strains.

Molecular investigation of a 401-nt NS3 genomic region of the magpie strain revealed the presence of proline at the 249 aa position of NS3 gene, a mutation related to increased viremia potential and virus transmission rates in corvids for L1 strains [20]. This mutation was also present in the other WNV L2 strains isolated in Greece [18,21]. All other WNV L2 strains isolated worldwide have histidine at this position and have not been related with major human outbreaks [18]. The NS3 genomic region sequence from the magpie isolate was deposited in GenBank under accession no. JN809471.

## Conclusions

This is the first report regarding extensive exposure of wild birds to WNV in Greece prior to the 2010 human outbreak. Findings of high virus neutralization titers in many samples suggests a possible recent exposure to WNV rather than cross reaction to other flaviviruses such as USUV. In addition, our employed RT- PCR designed to amplify a wide range of mosquito-borne flaviviruses did not give any positive result other than WNV. Resident corvids hunter-harvested in the epicenter of the outbreak have been exposed to WNV at least eight months before the first human cases were reported. Thus an active wildlife surveillance system for emerging infectious diseases would predict the mosquito-wild birds WNV circulation and the possible emergence under appropriate conditions that caused the major outbreak in humans. Genetic determinants of increased virulence were present in the WNV strain isolated from the magpie that further support this finding. However, different findings have also been reported regarding the NS3_249_ mutation suggesting that this mutation may not be sufficient to enhance virulence for any given WNV strain [22]. Thus, experimental infection studies and pathogenicity assessment will provide more solid conclusions. Furthermore, genetic variation was observed in the related Greek strains isolated from different hosts; this supports the hypothesis of the quasispecies structure and possible process of adaptation to local transmission of the virus [23].

Even though our study confirmed the presence of important genetic virulence markers in the magpie strain, clinical signs suggestive of encephalitis or dead birds were not reported from any of the hunters. The hunters had been briefed and were instructed to report any such observations. Wild birds in Greece do not seem to be susceptible to WNV even though the virus was able to cause a major human outbreak; this further supports the hypothesis that birds in Europe may have an innate immunity due to the ancestral co evolution and long association between WNV and avian hosts in the Old World [8].

Findings of WNV neutralizing antibodies in migratory hunter-harvested turtle doves (some juvenile and with high VNT titers) upon their arrival in resting areas of Greece during their autumn migration from breeding areas of central Europe to wintering areas of Africa, indicates exposure probably in the areas of their origin. This finding in addition to the molecular similarity of WNV strains isolated in Greece with strains isolated in previous years in Austria and Hungary [13,14] suggests avian species with similar migration traits as candidates for possible introduction of WNV L2 strain into Greece in previous years from central Europe. The detection of a WNV L2 infection in Italy in 2011, with the isolated strain being closer phylogenetically to the Hungarian and Austrian L2 strains rather than African strains supports this hypothesis [24], especially as Italy and Greece are stopover areas during autumn migration of various avian species from central Europe to Africa [25]. Of course, we cannot exclude the possibility of infection of migratory birds at the stopover areas or that an endemic circulation of WNV could have caused the outbreak after an amplification cycle due to favourable conditions present in the epicenter of the outbreak.

Results of this study strengthen the need for a continuous active serological and molecular surveillance system regarding WNV and other flaviviruses, which may provide timely information regarding virus introduction and circulation, further dispersion or introduction of new strains.

## Competing interests

The authors declare that they have no competing interests.

## Authors' contributions

PB and AG collected the samples, and created the map figure. ZD and CI prepared serum and tissue samples and performed the RNA extraction. GV, AT and LA carried out virological and molecular work. GV, CB, VS and LP conceived the study, selected and organized the employed protocols, carried out the sequencing studies and phylogenetic analysis and drafted the manuscript. All authors read and approved the final manuscript.
